# Omental Tissue-Mediated Tumorigenesis of Gastric Cancer Peritoneal Metastases

**DOI:** 10.3389/fonc.2019.01267

**Published:** 2019-11-18

**Authors:** Olga Kersy, Shelly Loewenstein, Nir Lubezky, Osnat Sher, Natalie B. Simon, Joseph M. Klausner, Guy Lahat

**Affiliations:** ^1^Laboratory of Surgical Oncology, Tel-Aviv Sourasky Medical Center, Tel Aviv-Yafo, Israel; ^2^Division of Surgery, Tel-Aviv Sourasky Medical Center, Tel Aviv-Yafo, Israel; ^3^Sackler Faculty of Medicine, Tel-Aviv University, Tel Aviv-Yafo, Israel; ^4^Institute of Pathology, Tel-Aviv Sourasky Medical Center, Tel-Aviv, Israel; ^5^College of Arts and Sciences, University of Virginia, Charlottesville, VA, United States; ^6^The Nikolas and Elizabeth Shlezak Cathedra for Experimental Surgery, Sackler Faculty of Medicine, Tel-Aviv University, Tel Aviv-Yafo, Israel

**Keywords:** omental tissue, gastric cancer, tumor microenvironment, peritoneal metastasis, exosomes

## Abstract

The peritoneal cavity, especially the omentum, is a common site for gastric cancer metastasis, representing advanced disease stage and poor prognosis. Here, we studied the effects of omental tissue on gastric cancer tumor progression *in vitro* and *in vivo*. Utilizing *in vitro* models, we found that omental tissue secreted factors increased gastric cancer cellular growth (by 30–67%, *P* < 0.05), motility (>8-fold, *P* < 0.05), invasiveness (>7-fold, *P* < 0.05) and chemoresistance to platinum-based chemotherapeutic agents (>1.2-fold for oxaliplatin and >1.6-fold for cisplatin, *P* < 0.05). Using a robust proteomic approach, we identified numerous molecules secreted into the omental tissue conditioned medium (CM) which may promote gastric cancer cellular aggressiveness (i.e., IL-6, IL-8, MMP9, FN1, and CXCL-5). Next, an *in vivo* xenograft mouse model showed an increased human gastric adenocarcinoma tumor volume of cells co-cultured with human omental tissue secreted factors; 1.6 ± 0.55 vs. 0.3 ± 0.19 cm^3^ (*P* < 0.001), as well as increased angiogenesis. Finally, exosomes were isolated from human omental tissue CM of gastric cancer patients. These exosomes were taken up by gastric cancer cells enhancing their growth (>8-fold, *P* < 0.01) and invasiveness (>8-fold, *P* < 0.001). Proteomic analysis of the content of these exosomes identified several established cancer- related proteins (i.e., IL-6, IL-8, ICAM-1, CCl2, and OSM). Taken together, our findings imply that the omentum play an active role in gastric cancer metastasis. The data also describe specific cytokines that are involved in this cross talk, and that omental tissue- derived exosomes may contribute to these unique cellular interactions with gastric cancer cells. Further studies aimed at understanding the biology of gastric cancer intra peritoneal spread are warranted. Hopefully, such data will enable to develop future novel therapeutic strategies for the treatment of metastatic gastric cancer.

## Introduction

Gastric adenocarcinoma is the fourth most common cancer and the second leading cause of cancer-related mortality worldwide (1). This high mortality rate is mainly a result of late diagnosis and limited therapeutic options despite considerable improvements in surgical capabilities and multidisciplinary care (2, 3). To date, surgery is the only potentially curative treatment for gastric cancer; however, more than two-thirds of patients have unresectable disease upon diagnosis (4). Peritoneal carcinomatosis, specifically, malignancy within the omentum, is a very common form of metastatic spread in gastric cancer patients, and disseminated peritoneal lesions are usually untreatable due to their high resistance to chemotherapy (5, 6). While the effect of omental tissue on omental metastasis of ovarian, pancreatic and colon cancer has been described in several studies (7–10), experimental data on gastric cancer omental metastasis are scarce.

The omentum is composed of adipocytes, endothelial cells, immune cells, stromal cells and aggregates of well-vascularized immune cells called “milky spots” (11). These cells secrete various adipokines which may play a role in gastric cancer tumor progression. It is possible that some of these adipokines are delivered through exosomes. Exosomes are 50–140 nm extracellular vesicles that can be released into the extracellular space from many cell types, including the omentum (12, 13). These exosomes contain proteins and RNA molecules that can be transferred to different recipient cells, thus affecting their biological behavior (14–16). Adipose tissue-derived exosomes have been reported to be implicated in obesity-related metabolic diseases, such as type 2 diabetes and non-alcoholic fatty liver disease (17–20). However, little is known about the involvement of adipose tissue-derived exosomes in tumorigenesis and, to the best of our knowledge, there are no data on the role of exosomes in the interaction between the omentum and metastatic epithelial cancer cells.

In this study, we show the *in vitro* and *in vivo* effects of human omental tissue-secreted factors on gastric cancer cellular growth, invasion, and resistance to chemotherapy. Furthermore, we demonstrate that these effects may be mediated, at least to some extent, through exosomes. Understanding the mechanisms of omental metastasis will hopefully lead to the discovery of potential molecular diagnostic markers and to novel targets for therapy.

## Materials and Methods

### Cell Culture

AGS, SNU-16, and N-87 human gastric adenocarcinoma cells were purchased from the American Tissue Culture Collection (ATCC). Cells were detected as *Mycoplasma*-free by PCR-based method (Hymicoplasma Detection Kit) and were cultured for no more than 20 passages between thawing and use in experiments. AGS cells were cultured in Dulbecco's modified Eagle's medium (DMEM) supplemented with 10% heat-inactivated fetal bovine serum (FBS) and 100 U/ml penicillin-streptomycin (Biological Industries Ltd, Beit Haemek, Israel). SNU-16 cells (grown in suspension) and N-87 cells were cultured in RPMI-1640 medium supplemented with 10% heat-inactivated FBS and 100 U/ml penicillin-streptomycin (Biological Industries). The cells were maintained in a humidified 5% CO_2_ atmosphere at 37°C (21). All cells treated with exosomes were grown in medium supplemented with 10% exosome-depleted FBS prior to the assay.

### Human Sample Collection and Conditioned Medium (CM) Preparation

This study protocol was approved by the Human Ethics Review Committee of the Israeli Ministry of Health and the Tel-Aviv Sourasky Medical Center. All subjects gave written informed consent in accordance with the Declaration of Helsinki. Fresh human omental tissue located outside the clean macroscopic surgical margins was harvested from patients with gastric cancer who were undergoing surgery. Inclusion criteria were operable gastric cancer, no evidence of peritoneal spread during surgery, no evidence of parenchymal involvement according to a pre-operative computerized tomographic scan, and a body mass index <30. No patients had metastatic disease, however, nodal status, the presence of lymphovascular invasion and level of differentiation differed among them (10). The omental tissues were harvested from 50 patients undergoing gastric cancer surgery. Each experiment was repeated at least three times and utilized a different sample each time. Adipose tissue explants were prepared as described elsewhere (22). Briefly, cultured omental tissue fragments (2–3 mm^3^, 100 mg/ml medium) were incubated at 37°C in medium (M199 + 10% FBS, 2 mM L-glutamine) and allowed to settle for 2 h. The medium was replaced, and the fragments were further incubated for 24 h in serum-free M199 (0.5% BSA). Under these conditions, the explants remain viable and functional for at least 48 h. The viability of adipose tissue explants after 24 h of incubation was also documented by the activity of lactate dehydrogenase (LDH) in the medium after 24 h. LDH activity in adipose tissue lysate was used as the positive control. The LDH values for all samples were within normal limits for control cells (12.6 ± 4.9%) in all experiments. The fragments were removed with tweezers, and the CM was transferred from the well to a clean tube and immediately utilized or quickly frozen (10 s) in liquid nitrogen and stored at −80°C. The medium in which the omental tissue cells explants were prepared and incubated (M199 regular medium-RM) prior to the collection of the CM was used as control (23).

### Exosome Isolation From the CM of Omental Tissue Explants

Omental tissue exosomes were extracted from the CM of human omental tissue explants cultured for 24 h in serum-free M199 media. Cell culture supernatants were collected and exosomes were isolated by differential centrifugation as described by Thery et al. (24). The pelleted exosomes were re-suspended in PBS and quantified using the Bradford assay (Bio-Rad).

### Cell Growth Assays

Cell proliferation was measured using the XTT cell proliferation kit (Biological Industries) according to the manufacturer's instructions. Briefly, 5,000 cells/well were plated in a 96-well plate and incubated with RM, CM or with 10 and 100 μg/ml exosomes for 24 h. For cell inhibition assay, the cells were incubated with RM or CM and increasing doses of oxaliplatin (50–100 μM) or cisplatin (5–200 μM) for 24 h for the AGS cells and 48 h for the N-87 cells (25, 26). For clonogenic assay, 100 viable AGS and N-87 cells were plated in a 6-well plate in RM or CM. The cells were incubated for 14 days at 37°C, and the formed colonies were fixed with paraformaldehyde (4%), stained with crystal violet (0.5%), and analyzed morphologically by quantifying the number of colonies formed per well using a microscope (10). All experiments were repeated three times for each cell line with CM from three different patients.

### Scratch Wound Healing Assay

Scratch wound healing assay was performed as described elsewhere using AGS cells (10, 23). Cells were seeded in a 6-well tissue culture plate and allowed to grow to ~95% confluence. The plates were scratched with a 200 μl pipette tip across the center of the well to create a straight line. The cells were washed twice with phosphate buffered solution (PBS) to remove any detached cells, and fresh RM, CM, or 10 μg exosomes was added to each well. Images were captured under the microscope at 0 and 24 or 48 h to assess the rate of gap closure.

### Migration and Invasion Assays

Transwell migration and invasion assays were conducted as described elsewhere (10, 23). Briefly, 5 × 10^4^ AGS or 1 × 10^5^ SNU-1 and 1 × 10^5^ N-87 cells suspended in RM (0.5 mL/chamber) were placed in the upper chamber and allowed to migrate into 24-well Transwell inserts with 8-mm pore size (BD Biosciences). The lower chamber was filled with 0.75 ml/well of RM or CM supplemented with 2% FBS. Invasion assays were conducted similarly using 24-well transwell inserts with 8 μm pore polycarbonate filters coated with a thin layer of 10 mg/ml growth factor reduced Matrigel (BD Biosciences). For exosome-induced migration, the lower chamber was filled with DMEM supplemented with 2% exosomes-depleted FBS and 10 μg/ml exosomes. In all cases, the cells were incubated for 16 h at 37°C. After incubation, the filters were fixed with 4% formaldehyde and stained with 0.2% crystal violet. Cells on the upper surface of the filters were removed by wiping with a cotton swab, and migratory and invasive activities were determined by counting the number of cells in three fields per well (magnification, ×100) in triplicates. The number of migrated or invaded cells was quantified by counting cells with the ImageJ 1.48v.Java image processing program.

### Apoptosis Assay

Apoptosis was measured using the Apoptosis Kit (MEBCYTO Apoptosis Kit, MBL) according to the manufacturer's recommendations. In brief, 1 × 10^6^ AGS and N-87 cells were incubated with omental tissue CM or RM and increasing doses of oxaliplatin (50–200 μM) or cisplatin (40–200 μM) for 24 h for AGS cells and 48 h for N-87 cells. The cells were trypsinized, washed, and resuspended in binding buffer. After the addition of Annexin V-FITC and PI to the cell suspension, the cells were incubated for 15 min in the dark and measured by FACS (BD FACS Canto II) using a single laser emitting excitation light at 488 nm (10).

### Cell Cycle Analysis

1 × 10^6^ cells were incubated with CM or RM for 24 h, trypsinized, centrifuged, and resuspended with 300 μl of cold PBS. The cells were fixed with 70% cold ethanol, treated with 5 μg of ribonuclease A (RNase A) and 10 μg of propidium iodide (PI), after which they were analyzed by FACS (10).

### Liquid Chromatography and Tandem Mass Spectrometry (LC-MS/MS)

CM from explants of human visceral fat and subcutaneous fat (*n* = 6) were collected and subjected to LC-MS/MS as described previously (10, 27).

### *In vivo* Animal Models

All animal procedures and care were approved by the Institutional Animal Care and Usage Committee (protocol # 2n-1-15). Animals received humane care as per the Animal Welfare Act and the NIH “Guide for the Care and Use of Laboratory Animals.” AGS (1 × 10^6^/0.1 mL PBS/mouse) cells were pre-incubated *in vitro* for 2 days with human omental tissue CM or non-conditioned medium prior to their subcutaneous injection into the flank of 6-week-old male athymic nude mice Foxn1^nu/+^ (*n* = 5/experiment). The mice were followed for tumor size, well-being, and body weight, and sacrificed when any tumor reached an average of 1.5 cm^3^ in its largest dimension. The tumors were resected, weighed, and frozen or fixed in formalin and paraffin-embedded for H&E staining and immunohistochemistry (IHC). Tumor volume was calculated using the following formula: tumor volume = L × W^2^ × π/6 (cm^3^) where L is the tumor's length and W is its width (10, 23).

### Immunohistochemistry (IHC)

IHC was performed with the Ventana Benchmark automated staining system (Ventana Medical Systems, Tucson, AZ) on 4-μm paraffin sections. The slides were deparaffinized in xylene and rehydrated through a graded series of ethanol concentrations. Tissue sections were stained using the following primary antibody: Ki67 (1:100, Spring Bioscience, CA); CD31 (1:100, Cell Marque, CA, USA); S100 (1:100, Cell Marque) and loaded into a Benchmark XT (Ventana Medical Systems Inc, Tucson, AZ) automated stainer. Primary antibodies were detected with the Ventana iVIEW DAB detection kit. Scoring of Ki67 and CD31 protein expression was interpreted independently by an expert gastrointestinal pathologist (SO). For quantification of the proliferation, the percentage of Ki67-positive nuclei was determined in five of the most proliferating areas within a tumor (“hot spots”) (×200 magnification, *n* = 10). To quantify angiogenesis, blood vessels/cells were counted in a representative high-power (×200) field. Blood vessel density was calculated as the mean ± SD of all counts (×200 magnification, *n* = 10) (10, 23).

### Cryogenic Transmission Electron Microscopy (Cryo-TEM)

Specimen preparations were prepared in a controlled environment vitrification system (CEVS) (28). Specimens were prepared at a constant temperature of 25°C. To prevent solvent evaporation and changes in solvent concentration, the specimens were prepared in a chamber at 100% relative humidity. Prior to specimen preparation, grids were plasma etched in a PELCO EasiGlow glow-discharger (Ted Pella Inc., Redding, CA) to increase their hydrophilicity. A drop of the sample was pipetted onto a carbon-coated perforated polymer film, supported by a 200 mesh TEM grid (Ted Pella Inc., Redding, CA, USA) held by tweezers inside the chamber. The drop was thinned into a film <300 nm thick, by blotting away excess solution with a filter paper wrapped on a metal strip. The grid was then plunged (dropped mechanically) into liquid ethane at its freezing point (−183°C) cooled by LN2 at its boiling point (−196°C) (29, 30). Cryogenic transmission electron microscopy (cryo-TEM) imaging was performed on a Thermo-Fisher Talos F200C, FEG-equipped high resolution-TEM, operated at 200 kV. Specimens were transferred into a Gatan 626.6 cryo-holder and equilibrated below −170°C. Micrographs were recorded by a Thermo-Fisher Falcon III direct detector camera, at a 4 × 4 k resolution. Imaging was performed at a low dose mode of work to minimize the exposure of the imaged area to electrons. Images were acquired using the Tem Imaging and Acquisition (TIA) software.

### Nanoparticle Tracking Analysis (NTA)

Size distribution analysis of exosomes based on Brownian motion was assayed by NanoSight LM20 (NanoSight, Amesbury, UK). Briefly, the exosomal fraction was diluted 1:1,000 with PBS at 23°C and size dispersion was measured. A video of 30–60 s with a frame rate of 30 frames/s was taken, and particle movement was analyzed by NTA software (version 2.3, NanoSight) (31).

### Exosome Labeling

Exosomes labeling was performed as described by Hazan-Halevy et al. (31). Exosomes were diluted in PBS and labeled with PKH-67 green fluorescent cell linker cell membrane labeling dye (4 μl) (Sigma-Aldrich) was added to 1 ml of Diluent C before being added to the exosomes. Samples were mixed gently for 5 min followed by the addition of 1% BSA (2 mL) to bind the excess dye. The samples were then transferred to 100 kDa Amicon (Merck Millipore, Merck KGaA, Darmstadt, Germany) and centrifuged at 4,000 × g for 30 min. The samples were washed three times with 5 ml of PBS before being transferred to a 7 kDa Zeba spin desalting column (Thermo Scientific, Pierce Protein Biology Products) to remove the remaining free dye.

### Exosomes Internalization Assays

Internalization was measured by confocal microscopy and flow cytometry analysis. For flow cytometry, AGS cells (5 × 10^5^) were incubated for the indicated times at 37°C with PKH-67 labeled- exosomes (2 μg) and resuspended in 500 μl of exosomes-depleted cell media. The samples were transferred to ice and washed three times with cold PBS. Data were acquired by FACS Canto II with Diva software (Becton Dickinson, Franklin Lakes, NJ). Data analysis was performed using FlowJo software (Tree Star, Inc. OR). For confocal microscopy analysis, the AGS cells were grown to 50% confluence on 13 mm coverslides in a 24-well plate and incubated for the indicated times at 37°C with PKH-67-labeled exosomes (2 μg). The cells were then washed twice with PBS and fixed with 4% PFA for 10 min. The nuclei were stained with Dapi, and the slides were viewed under a Zeiss LSM 700 confocal microscope. For exosome internalization inhibition, AGS cells were treated with 10 ng/mL Heparin (Sigma-Aldrich), 80 μM Dynasore (Sigma-Aldrich) for 30 min or cultured in serum free DMEM media supplemented with 0.1% BSA and 20 mM Hepes in the presence of 2 μM Simvastatin (Sigma-Aldrich) for 16 h (31). PKH-67 labeled omental tissue exosomes were incubated with untreated or treated cells for 3 h at 37 or 4°C and internalization was measured by flow cytometry and confocal analysis as described above.

### Western Blot Analysis

Cells and exosomes pellets were lysed with RIPA lysis buffer (Thermo Scientific, Pierce Protein Biology Products). Proteins (6 μg) were electrophoresed on SDS-PAGE. Western blotting analyses were performed using the following antibodies: mouse monoclonal anti-human CD81 (Santa-Cruz Biotechnology; SC-166029) and mouse monoclonal anti-human CD63 (Santa-Cruz Biotechnology; SC-5275).

### Human Cytokine Antibody Array

The content of exosomes proteins were screened by the Human Cytokine Antibody Array C1000 (RayBio, USA). Exosomes protein lysates were obtained from a pool of four human omental tissue specimens and four human subcutaneous fat specimens using 2× cell lysis buffer (RayBio, USA) and quantified using Bradford assay (Bio-Rad). Each array membrane was loaded with 50 μg of each exosomes pool lysate, and the array was performed according to the manufacturer's instructions. Expression of each protein was represented in duplicate on each array membrane. Duplicate dots identifying each protein were quantified by ImageJ software.

### Statistical Analysis

Statistical analysis was performed using GraphPad Prism^TM^ software. Numerical values of cell culture and mouse xenograft data were analyzed using Student's *t*-test for significance. Results are expressed as means ± SD. The indicated number of experiments (n) refers to the experiments performed with omentum from different patients (10). A *P* ≤ 0.05 was considered as being significant.

## Results

### Omental Tissue Induces Gastric Cancer Cell Growth

**Figure 1:**
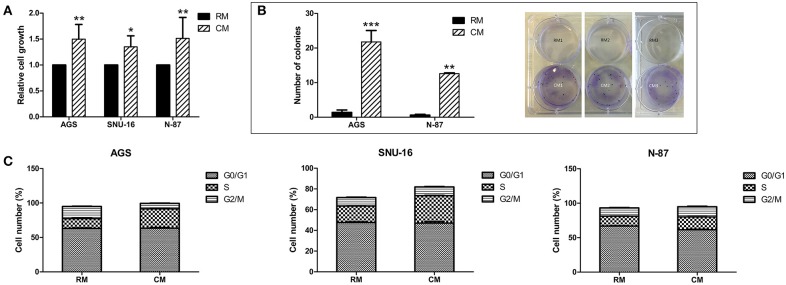
Omental tissue CM enhances gastric cancer cell growth. **(A)** XTT assay showing significant increased proliferation (*P* < 0.05) of gastric cancer cells co-cultured with omental tissue CM; *n* = 4 **(B)** Omental tissue CM enhanced gastric cancer cell clonogenicity (*P* < 0.01). Left panel graphs represent the average of five repeated experiments ± SD, Right panel depicts representative images of AGS (left and middle rows) and N-87 (right row) colonies; *n* = 5 **(C)** Omental tissue CM enhanced S-phase population in gastric cancer cells; a more prominent effect was seen in AGS and SNU-16 (*P* < 0.05) cells than in N-87 cells; *n* = 6. *P < 0.05; **P < 0.01; ***P < 0.001.

We utilized three human gastric cancer cell lines to assess the potential effects of human omental tissue CM on gastric cancer cell cycle and growth. AGS derived from primary gastric adenocarcinoma, N-87 derived from liver metastasis, and SNU-16 derived from gastric cancer malignant ascites were incubated with conditioned medium (CM) or regular medium (RM). We demonstrated that omental tissue CM significantly increased the proliferation of all gastric cancer cell lines compared to RM using an XTT proliferation assay (*P* < 0.05; Figure 1A). In addition, we used a colony formation assay to investigate the effect of omental tissue CM on the clonogenicity of N-87 and AGS cell lines. As depicted in Figure 1B, the colony formation capacity of these cells was significantly increased after 14 days of incubation with omental tissue CM compared to RM (*P* < 0.01). We next evaluated the potential effect of omental tissue CM on cell cycle progression. AGS, SNU-16, and N-87 cells treated with omental tissue CM underwent a significant increase in the S phase population compared with the control RM-treated cells (67, 65, and 28%, respectively, *P* < 0.05; Figure 1C). Taken together, these data suggest that omental tissue CM promotes gastric cancer cellular growth and proliferation, possibly explained, at least in part, by fat-induced cell cycle deregulations.

### Omental Tissue Increases Gastric Cancer Cell Migration and Invasion

**Figure 2:**
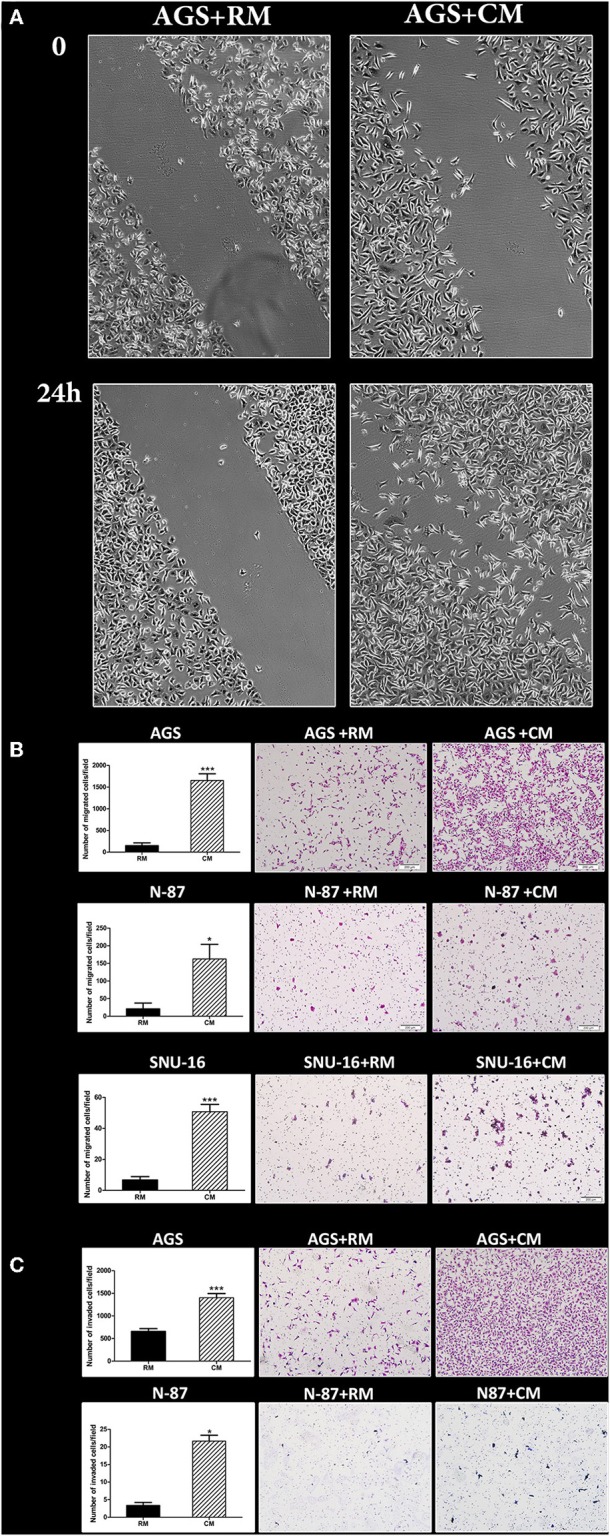
Omental tissue CM augments gastric cancer cell migration and invasion. **(A)** Scratch wound healing assay displaying the effect of omental tissue CM on AGS cells migration; Scale bar = 200 μm; **(B)** Transwell assays demonstrating the effects of omental tissue CM on gastric cancer cell migration (*P* < 0.05); **(C)** Matrigel invasion chamber assay showing a significant increase in invasion of AGS and N-87 cells. Omental tissue CM was used as chemoattractant (*P* < 0.05); Left panel graphs represent the average of 5 repeated experiments ± SD, and the right panel depicts representative images (magnification, ×100). *P < 0.05; ***P < 0.001.

We next evaluated the effect of omental tissue CM on gastric cancer cell migration and invasion. AGS cells underwent a scratch wound healing assay following pre-treatment with omental tissue CM or RM for 24 h. Scratch assay was not applicable for the N-87 cell line due to its nature to grow in dense patches. Figure 2A depicts the marked increase in the migration of AGS cells when treated with omental tissue CM. We utilized the modified Boyden chambers system to evaluate the chemotactic potential of omental tissue on gastric cancer cellular migratory potential and invasiveness. The Boyden chamber system consists of transwell inserts of 8 micron pore size membrane with or without a thin layer of extracellular matrix (Matrigel Basement Membrane Matrix). The layer occludes the pores of the membrane thus blocking non-invasive cells from migrating through the membrane. In contrast, invasive cells are able to invade through the matrix and the 8 micron membrane pores. Gastric cancer cells were grown on transwell inserts with or without Matrigel and assessed for their ability to invade or migrate, respectively, toward omental tissue CM for 16 h. Omental tissue CM significantly increased migration of both AGS and N-87 cells (>7-fold and >8-fold, respectively) and invasion (>2-fold; >7-fold, respectively) (*P* < 0.05; Figures 2B,C). As depicted in Figure 2B, omental tissue CM also increased the migration of SNU-16 cells (>7-fold; *P* < 0.001). However, due to its inability to adhere, we were not able to evaluate the potential effect of the omentum on SNU-16 invasion capacity using the transwell assay. These results demonstrated that omental tissue-secreted factors increased gastric cancer cell motility and invasiveness, implying that it may also play a role in the chemotaxis of gastric cancer cells.

### Omental Tissue CM Augments Gastric Cancer Cell Chemoresistance

**Figure 3:**
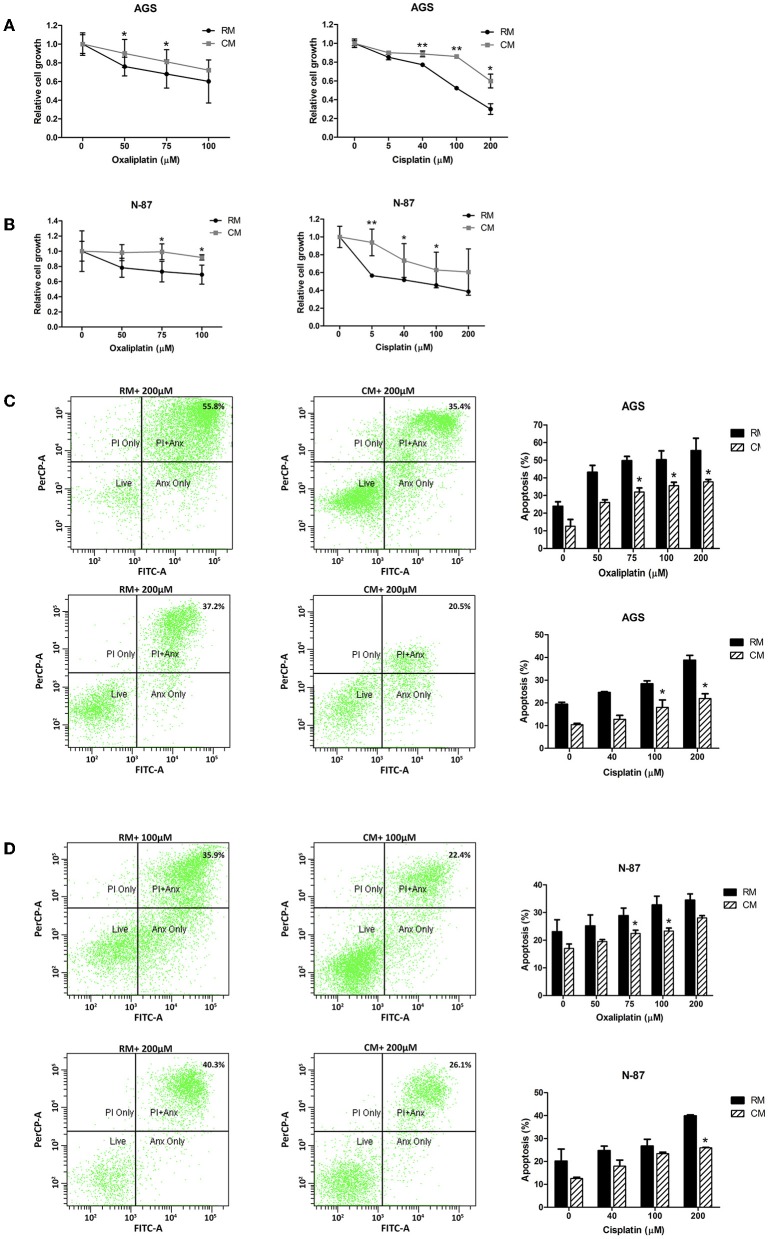
Omental tissue CM augments gastric cancer cells resistance to chemotherapy. **(A,B)** Increased survival of oxaliplatin and cisplatin -treated AGS **(A)** and N-87 **(B)** gastric cancer cells following co-culture with omental tissue CM (*P* < 0.05), *n* = 4; **(C,D)** FACS analysis using Annexin-V/PI demonstrating a significant reduction in oxaliplatin and cisplatin -induced apoptosis of AGS **(C)** and N-87 **(D)** cells co-cultured with omental tissue CM (*P* < 0.05), *n* = 5. *P < 0.05; **P < 0.01.

While chemotherapy plays a significant role in the treatment of both local and metastatic gastric cancer, its efficacy is often limited by chemoresistance (32). We hypothesized that gastric cancer cells cultured with omental tissue CM would show resistance to platinum-based chemotherapeutic agents, the mainstay of anti-gastric cancer therapy (33). To test this hypothesis, AGS and N-87 cells cultured with omental tissue CM or control RM were treated with increasing doses of cisplatin (5–200 μM) or oxaliplatin (50–100 μM), and chemosensitivity was determined by XTT assay. The AGS and N-87 cells that had been cultured with omental tissue CM demonstrated a significantly higher survival rate after platinum treatment compared with the control medium (>1.2-fold for 75 μM of oxaliplatin for both cells, and >1.6-fold for 5 μM of cisplatin for N-87 cells and 100 μM of cisplatin for AGS cells, *P* < 0.05; Figures 3A,B). Next, we conducted FACS analyses using Annexin V-FITC and PI staining. The AGS and N87 cells were treated with increasing doses of oxaliplatin (50–200 μM) or cisplatin (40–200 μM). As depicted in Figures 3C,D, there was a significant decrease in the percentage of apoptotic cells in both of those gastric cancer cell lines when cultured in omental tissue CM compared to RM; 1. AGS: 37 vs. 55.4% (*P* < 0.05) and 21.9 vs. 39% (*P* < 0.05) for oxaliplatin and cisplatin, respectively (Figure 3C), and 2. N-87: 23.3 vs. 32.7% (*P* < 0.05) and 26.3 vs. 40% (*P* < 0.05) for oxaliplatin and cisplatin, respectively (Figure 3D).

These *in vitro* data suggest that omental tissue CM augment gastric cancer tumoral growth and survival as well as increased cellular motility and invasiveness. These results also suggest that omental tissue has a role in chemoresistance of gastric cancer cells.

### Human Omental Tissue Promotes Gastric Cancer Xenograft Growth in Nude Mice

**Figure 4:**
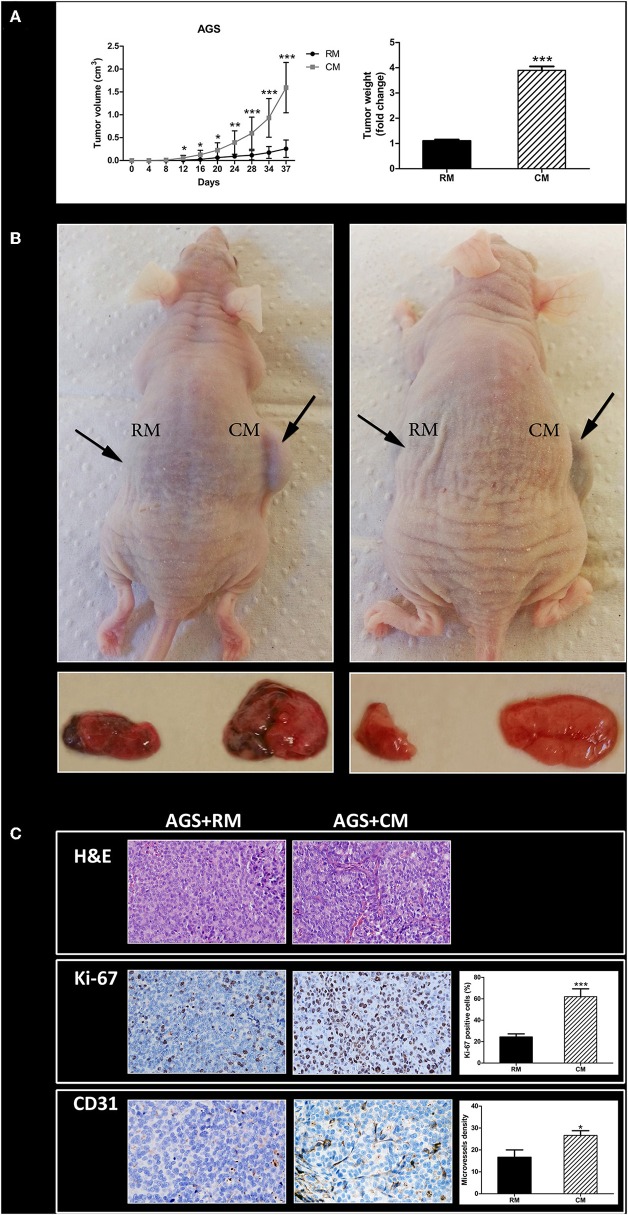
Omental tissue promotes tumor growth *in vivo*. **(A)** Growth and weight of AGS tumors was facilitated in mice after incubation with omental tissue CM (*n* = 15). Graphs represent the average of three repeated experiments ± SD (*P* < 0.005); **(B)** Images of representative tumor and mice; **(C)** Human omental tissue CM increase the proliferation (Ki-67) and microvessel density (CD31) of mice xenografts. Representative images are shown on the left (H&E, ×200; Ki-67, ×200; CD31, ×200). Immunohistochemistry (IHC) quantification is shown on the right. *n* = 10 in tissues of each site. *P < 0.05; **P < 0.01; ***P < 0.001.

**Figure 5:**
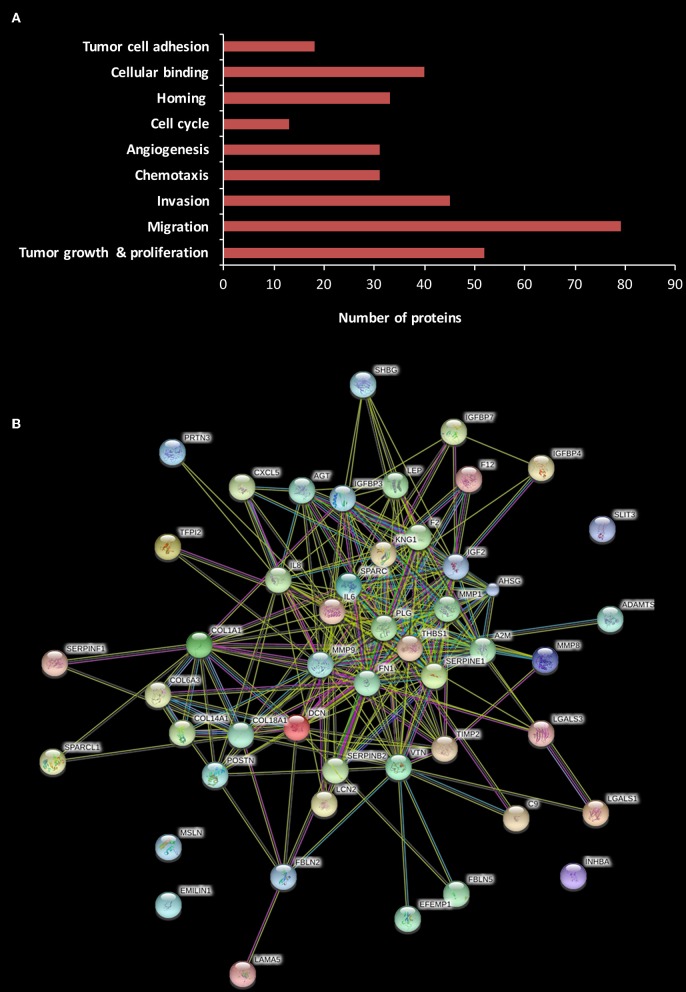
Human omental tissue secretome includes various pro-tumorigenic factors. **(A)** Functional annotation of differentially expressed proteins in omental tissue CM compared to SC fat CM. The stacked bar chart shows the number of proteins in each category; **(B)** Protein-protein interactions network of 52 proteins related to tumor growth and proliferation constructed using STRING analysis.

Next, we sought to evaluate whether human omental tissue-secreted factors affect gastric cancer tumor growth *in vivo*. For that purpose, we used a human gastric cancer xenograft model in nude mice (10). The AGS gastric cancer cells were co-cultured *in vitro* with human omental tissue CM or control RM before injected subcutaneously into the flank of nude mice. As shown in **Figure 5**, AGS cells co-cultured with omental tissue CM grew faster than the control tumors that were growing in the contralateral flank: tumor volumes were 1.6 ± 0.55 vs. 0.3 ± 0.19 cm^3^ (*P* < 0.001; Figure 4A). The mean tumor weight of the omental tissue CM-treated cells was almost 4-fold higher than the weight of the control AGS cells co-cultured with non-conditioned RM, 0.37 ± 0.16 vs. 0.09 ± 0.01 gr, respectively (*P* < 0.001; Figure 4A). Figure 4B depicts representative mice and the tumors harvested from both treatment groups.

In order to examine the extent of xenograft vascularization and proliferation, tumor sections from each treatment group were subjected to H&E staining (in order to confirm malignancy; Figure 4C) and then to IHC staining using markers of microvessel density (CD31) and tumor cell proliferation (Ki-67). The scores of Ki-67 in tumors pre-treated with omental tissue CM and in tumors pre-treated with RM were 63 ± 16.43 and 25 ± 8.36%, respectively (*P* < 0.001). In addition, IHC staining of CD31 demonstrated increased microvessel density in tumors treated with CM compared with the control RM treated cells, 26.66 ± 5.16 vs. 16.66 ± 9.57, respectively (*P* < 0.05; Figure 4C).

These data strongly support our *in vitro* experiments by showing that omental tissue CM contributes to gastric cancer xenograft growth *in vivo*.

### Human Omental Tissue Secretome Includes Various Pro-tumorigenic Factors

Liquid Chromatography and Tandem Mass Spectrometry (LC-MS/MS) was used to portray the secretome of the omentum. As previously described by others, subcutaneous (SC) fat was selected as the control (34). Based on the findings of our *in vitro* and *in vivo* experiments, we chose to evaluate the secretome of the omental tissue (as a whole) rather than to focus on one of its specific cellular components (i.e., adipocytes, endothelial cells, etc.). We analyzed the identified proteins using Ingenuity Pathways Analysis (IPA) based on their different cellular location, i.e., cytoplasm, nucleus, plasma membrane, and extracellular space. The LC-MS/MS analysis identified 800 proteins, of them 194 were extracellular. We further analyzed these 194 proteins according to their cellular and molecular function with the following results: 52 were related to tumor growth and proliferation, 79 to cellular migration, 45 to cellular invasion, 31 to chemotaxis, 44 to angiogenesis, 13 to cell cycle, 33 to cell homing, and 40 to cellular binding, while 18 were involved in tumor cell adhesion (Figure 5A).

We then explored specific proteins which had been reported to play a role in gastric cancer development and progression (35–41), and the results are depicted in Table 1. A critical step in the formation of gastric cancer omental metastasis is the attachment of tumor cells to the omentum. Our analysis identified a list of seven proteins specifically associated with homing and binding/adhesion of gastric cancer cells: fibronectin 1 (FN1), elastin microfibril interface-located protein 1 (EMILIN-1), interleukin-6 (IL-6), interleukin-8 (IL-8), thrombospondin-1 (THBS-1), vitronectin (VTN), and C-X-C chemokine ligand-5 (CXCL-5). All seven proteins were significantly up-regulated in the omental tissue compared to SC fat (*P* < 0.05). Interestingly, STRING analysis for the 52 proteins related to growth and proliferation demonstrated that most of these proteins were clustered around three of those proteins: THBS-1, FN-1, and IL-6, as well as plasminogen (PLG) (Figure 5B).

**Table 1:** Gastric cancer-associated proteins identified by LC-MS/MS analysis of human omental tissue (OF) vs. subcutaneous fat (SC).

**Bio function**	**Function annotation**	**Molecules**
Cellular growth and proliferation	Proliferation of tumor cells	ADAMTS4, COL6A3, CXCL5, IL6, IL8, MIF, MMP1, MMP8, MMP9, MSLN, PLG, THBS
Cellular movement	Migration of cells	MMP1, MMP8, MMP9, PLG, SERPINA1, TGFB1, THBS1, TIMP1, TIMP2
Cellular movement	Invasion of cells	FN1, IL6, IL8, SERPINA1, STC1, THBS1, TIMP1, TIMP2
Cellular movement	Chemotaxis of cells	CXCL5, FBN1, FN1, IL6, IL8, MIF
Angiogenesis	Angiogenesis of tumor	ECM1, FN1, IL6, IL8, SERPINE1
Cell cycle	Mitogenesis	APOE, IGF2, IGFBP3
Cellular movement	Homing of cells	CXCL5, FN1, IL6, IL8, THBS1, VTN
Cellular movement	Binding of cells	FN1, THBS1, VTN
Cellular movement	Adhesion of tumor cells	EMILIN1, FN1, THBS1, VTN

The LC-MS/MS data demonstrated that the omentum secreted numerous proteins with an established role in cancer progression and metastasis. These probably play a role in the phenotypic changes observed in our *in vitro* and *in vivo* experiments. Further evaluation of these proteins may enable the characterization of specific molecular pathways related to the crosstalk between gastric cancer and the omentum.

### Structural and Biochemical Characterization of Omental Tissue-Derived Exosomes

**Figure 6:**
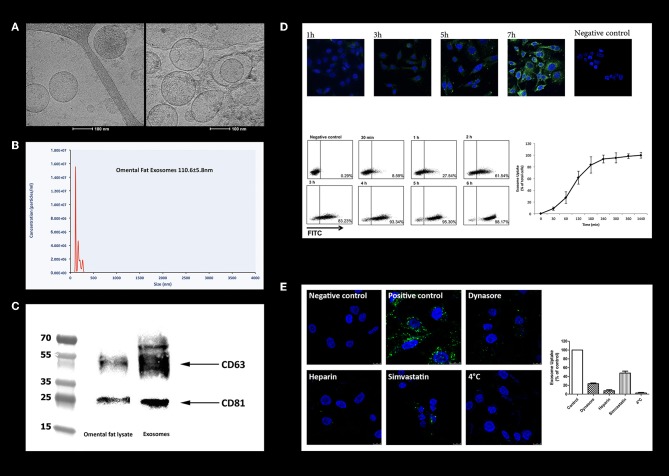
Characterization of human omental tissue-derived exosomes. **(A)** Exosomes were isolated from the CM of human omental tissue explants and analyzed by cryogenic transmission electron microscopy (Cryo-TEM); Scale bar = 100 nm. **(B)** Measurement of omental tissue exosomes diameter by nanoparticle tracking analysis system; **(C)** Western blot analysis of the exosome markers CD81 and CD63. Representatives of three independent experiments are shown; **(D)** PKH-67-labeled omental tissue exosomes were incubated with AGS gastric cancer cells, reaction was stopped at different time points (1, 3, 5, and 7 h) and cells were analyzed by confocal microscopy (upper panel). The cell's nucleus was stained with Dapi. PKH-67 labeled omental tissue exosomes were incubated for the indicated time points (30 min, 1, 2, 3, 4, 5, and 6 h) with AGS gastric cancer cells. Internalization was measured by flow cytometry (lower panel). Negative control-AGS cells with no addition of labeled exosomes. The data are presented as mean ±SD of four independent experiments; **(E)** AGS cells were pre-treated with 80 μM Dynasore or 10 ng/mL Heparin for 30 min or with 2 μM Simvastatin for 16 h. PKH-67 labeled omental tissue exosomes were incubated with untreated (positive control) or treated cells for 3 h and uptake was measured by flow cytometry analysis (right panel). The data are presented as the mean percent of cells that uptake labeled exosomes ± SD of three independent experiments; PKH-67 labeled omental tissue exosomes were incubated with untreated (positive control) or treated cells (Dynasore, Heparin or Simvastatin) and internalization was measured by confocal microscopy (left panel). Scale bar is 10 μm.

To investigate whether omental tissue-derived exosomes contribute to the tumorigenesis of gastric cancer cells, we first purified exosomes from the CM of human omental tissue explants utilizing a classic exosome isolation protocol using ultracentrifugation (24). We next examined them using a cryogenic transmission electron microscope (Cryo-TEM). The electron microscope imaging revealed the presence of spherical particles round in shape with dimensions of 85–150 nm. The images clearly show the phospholipid bilayer structure of the exosome membrane (Figure 6A). The number and size distribution of the exosomes was quantified by nanoparticle tracking analysis which demonstrated a homogeneous population of omental tissue exosomes with a modal diameter of 110.6 ± 5.8 nm and exosome concentration of 5.08e + 008 ± 2.38e + 007 /ml (Figure 6B). The expression of the exosomes proteins markers (24) was characterized by western blotting of purified exosomes, which revealed enrichment in CD81 and CD63 compared to whole-cell lysate of omental tissue (Figure 6C). These results confirmed that omental tissue-derived exosomes were successfully extracted from the CM of human omental tissue explants.

### Human Omental Tissue-Derived Exosomes Are Internalized by Gastric Cancer Cells

Exosomes must be taken up by cells in order to exert effects on them. We incubated PKH-67-labeled omental tissue-derived exosomes with AGS cells at different time points and followed their uptake by confocal microscopy analysis. We observed exosomes internalization as early as 30 min following incubation. A higher accumulation of exosomes inside the cells was observed in longer incubation times (Figure 6D). We next performed quantitative flow cytometry of AGS cells incubated with PKH-67-labeled exosomes and measured the fluorescence intensity at different time points. As depicted in Figure 6D, 8.59% of exosomes were taken up by the cancer cells after 30 min of incubation, rising to 98.17% after 6 h of incubation. These results confirmed the rapid uptake of omental tissue-derived exosomes by gastric cancer cells.

### Internalization of Human Omental Tissue-Derived Exosomes Is Mediated by Several Endocytosis Pathways

We next explored the internalization pathway of omental tissue exosomes. Exosomes are usually taken up by cells in a variety of endocytic pathways, including clathrin-dependent endocytosis, and clathrin-independent pathways such as caveolin-mediated uptake, macropinocytosis, phagocytosis, and lipid raft mediated internalization (42, 43). First, to rule out whether omental tissue exosomes are internalized through direct plasma membrane fusion (non-energy dependent process) or via endocytosis, we incubated PKH-67 labeled exosomes with AGS cells at 4°C. We observed that incubation at 4°C inhibited 100% of the uptake, suggesting an energy-dependent process consistent with an endocytic pathway (Figure 6E). We next used several chemical inhibitors to block specific endocytoic up-take pathways. AGS gastric cancer cells were incubated with Dynasore, an inhibitor of dynamin-dependent endocytosis (44), Heparin, a heparan sulfate (HS) analog, or cultured in the presence of Simvastatin, an inhibitor of cholesterol synthesis (45). PKH-67 labeled omental tissue exosomes were added to the treated cells for 3 h. The internalization of exosomes was quantified by flow cytometry and analyzed by confocal microscopy. Uptake of omental tissue exosomes was significantly inhibited by Heparin (93 ± 4.2% inhibition) and to a lesser extent by Dynasore (76 ± 2.1% inhibition) and Simvastatin (53 ± 7.1% inhibition; Figure 6E).

These results suggest that several pathways involving heparan sulfate proteoglycans (HSPGs), dynamin tyrosine kinase and cholesterol, mediate endocytosis of omental tissue exosomes by AGS cells.

### Human Omental Tissue–Derived Exosomes Promote Cancer Cell Proliferation and Motility

**Figure 7:**
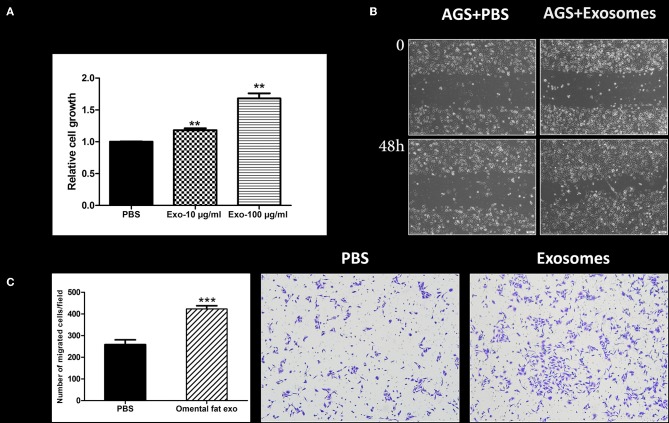
Human omental tissue-derived exosomes promote gastric cancer cell growth and motility. **(A)** AGS cells were treated with PBS, 10 or 100 μg/ml omental tissue exosomes in serum free medium. XTT assay was performed after 48 h of treatment (*P* < 0.01). The data are presented as mean ± SD of five independent experiments; **(B)** Scratch wound healing assay showing the effect of omental tissue exosomes on AGS gastric cancer cell motility; Scale bar = 200 μm; **(C)** Transwell assay depicting the effects of omental tissue exosomes on cell migration (*P* < 0.001). Left panel graph represents the average of three repeated experiments ± SD, and the right panel depicts representative images (magnification, ×100). **P < 0.01; ***P < 0.001.

Next, we sought to appraise whether omental tissue-derived exosomes take part in the potential crosstalk between the omentum and gastric cancer cells. As proof of concept, we focused on cellular proliferation and motility. Our results demonstrated that omental tissue-derived exosomes significantly increased gastric cancer cellular proliferation in a dose-dependent manner compared to the control non-treated cells (*P* < 0.05; Figure 7A). We next assessed the effect of omental tissue-derived exosomes on cellular migration. Wound healing assays showed that omental tissue-derived exosomes increased the migration of AGS cells (Figure 7B). To support this observation, we also used the modified Boyden chambers assay and, similarly, omental tissue-derived exosomes significantly increased the migration (>2-fold) of AGS cells compared with the control non-treated cells (*P* < 0.001; Figure 7C).

Our findings indicate that omental tissue secretes exosomes are taken up by gastric cancer cells and that these exosomes promote gastric cancer cellular growth and motility. These data suggest that the pro-tumorigenic effects that are induced by the omentum are mediated, at least in part, by exosomes. However, further study is needed to establish their potential role in the interaction between gastric cancer cells and the omentum.

### Human Omental Tissue-Derived Exosomes Express Various Pro-Tumorigenic Cytokines

**Figure 8:**
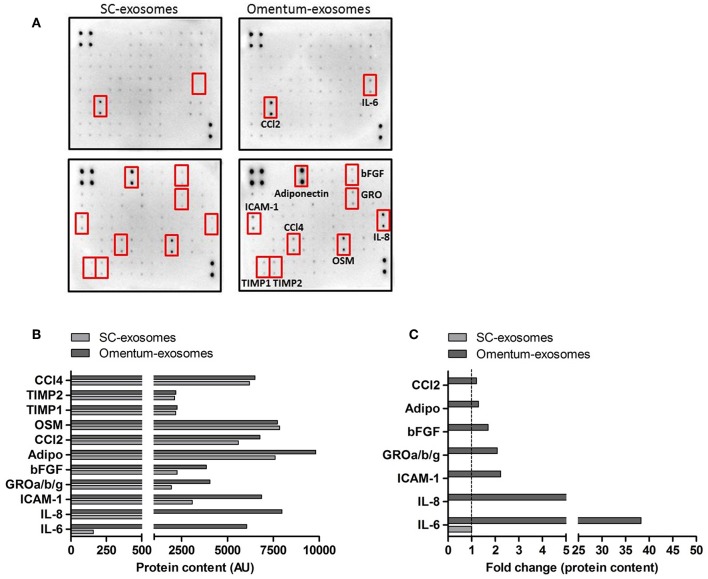
Human omental tissue-derived exosomes express various pro-tumorigenic cytokines. **(A)** Human cytokine antibody arrays used for the detection of cytokines obtained from a pool of four SC fat exosomes (left) and four omental tissue derived exosomes (right); **(B)** The intensities of the dots representing the cytokines highlighted by red boxes are expressed in arbitrary units (AU); **(C)** Data presented as fold change (FC) of proteins in omental tissue derived exosomes relative to SC fat exosomes.

In order to identify potential omental tissue-derived exosomal proteins that may be responsible for the observed effects on recipient gastric cancer cells, we performed a human cytokine antibody array on omental tissue-derived exosomes and SC fat-derived exosomes. The C1000 antibody array detects the levels of 120 different human cytokines. The expression levels of interleukin-6 (IL-6), interleukin-8 (IL-8), intercellular adhesion molecule-1 (ICAM-1), growth related oncogene (GRO), Basic fibroblast growth factor (bFGF), adiponectin and C-C Motif Chemokine Ligand 2 (CCl2) were increased in omental tissue-derived exosomes compared to SC fat exosomes (FC = 38.28, 8.6, 2.23, 2.08, 1.7, 1.29, and 1.2 respectively; Figure 8). In contrast, the expression level of oncostatin-M (OSM), tissue inhibitor matrix metalloproteinase-1 (TIMP1) and 2 (TIMP2) and C-C Motif Chemokine Ligand 4 (CCl4) were comparable between both exosome types. Interestingly, some of these cytokines had been reported as gastric cancer associated cytokines promoting gastric cancer proliferation, migration and survival and few of them were specifically associated with peritoneal metastasis (46–50). We compared our list of identified cytokines to the exosome and extracellular vesicles database ExoCarta and EVpedia (51, 52) and found that these proteins were not previously reported in adipose tissue exosomes.

The cytokine antibody array data suggest that omental tissue-derived exosomes contain various established cancer associated proteins. These proteins may induce the functional changes observed in our *in vitro* experiments in a paracrine or endocrine manner.

## Discussion

Intrinsic cancer cellular genetic alternations as well as the tumor microenvironment play a role in cancer progression and metastasis. Despite numerous studies that demonstrate an association between visceral fat and epithelial cancer progression, the exact mechanisms are poorly understood (53, 54). Understanding the molecular interactions between adipose tissue and cancer cells may enable us to detect potential targets for both prevention and treatment of intra-peritoneal spread. Since the omentum is a common metastatic site for gastric cancer as well as for other gastrointestinal epithelial cancers, we assumed that it has an active role in the homing and proliferation of tumor cells. The present study describes the pro-tumorigenic effects of omental tissue on gastric cancer cells, characterizes specific molecules which might be involved in this cellular interaction, and also suggests a potential role of exosomes in omental tissue-induced cancer progression.

Our data suggest that omental tissue cells secrete various factors, as well as exosomes, which increase gastric cancer cellular aggressiveness. Similarly, two recent studies described the role of adipose tissue stromal cells in the proliferation and invasion of gastric cancer cells (55, 56). The authors showed that adipose tissue stromal cells and adipocytes in particular increase gastric cancer cellular growth and invasion. Their findings imply that these phenotypic changes are possibly affected by MAPK activation in a COX-2-independent way (55) and through the PI3K/Akt signaling pathway (56). In addition, Cao et al. have shown that omental milky spots are a congenial microenvironment for peritoneal free gastric cancer cells to migrate, survive, and establish cell cluster-type metastases through the CCL22-CCR4 axis (57).

Unlike these earlier reports, we now sought to evaluate the omentum as a whole unit rather than to study its various components. For that purpose, we utilized an adipose tissue ex-plant culture system of human omental tissue as our experimental model. This system enabled us to recapitulate the physiological *in vivo* crosstalk between the various cell types of the omentum and gastric cancer cells. Initially, we demonstrated that omental tissue promotes gastric cancer cellular proliferation and increases the proportion of cells in the S-phase, indicating that factors secreted by the omentum possibly regulate the G1/S checkpoint. We also showed that omental tissue augments migration and invasion, the hallmarks of cancer metastasis. As such, these fat-induced protumorigenic effects may explain why the disseminated gastric cancer cells preferentially develop metastatic foci in the omentum, which provides a suitable scaffold upon which cancer cells can proliferate and thrive.

Despite advances in systemic and regional therapeutic modalities for gastric cancer peritoneal spread, patient outcome is poor, mostly due to drug resistance (5). While chemoresistance can be associated with genetic alterations within cancer cells, recent studies have proposed that it is also affected by the tumor microenvironment (58, 59). In that context, our results suggest that omental tissue CM may confer gastric cancer cellular resistance to platinum-based chemotherapeutic agents. We showed that omental tissue CM increased the survival rate of oxiliplatin- and cisplatin-treated gastric cancer cells. Furthermore, it prevented chemotherapy-induced apoptosis. Cytokines and growth factors exhibit key functions in chemoresistance by maintaining the activation of various survival-related signaling pathways. We assume that adipokines secreted by the omentum may similarly affect the chemoresistance of gastric cancer cells. Such adipokines include IL-6 and IL-8, both of which were increased in our omental tissue secretome. Interestingly, they were both included in a signature of 11 cytokines that could identify gastric cancer patients with poor prognosis when treated with standard chemotherapy (60). The potential role of these two adipokines, secreted by omental adipocytes and neutrophils, was also reported in relation to ovarian cancer omental spread (9, 61).

A subcutaneous (SC) gastric cancer model was used to evaluate whether a short and non-continuous exposure of gastric cancer cells to the omental tissue CM will increase *in vivo* tumor growth. As expected, the reliability of the pro-tumorigenic effects of omental tissue *in vitro* was confirmed by the *in vivo* experiments. Our results demonstrated that even a relatively short exposure of gastric cancer cells to omental tissue-secreted factors significantly increased tumor growth. We had previously shown that a short exposure of pancreatic cancer cells to omental tissue CM induced genetic alternations, indicating that omental tissue-secreted factors may induce cellular reprogramming in cancer cells and promote their aggressiveness (10). Combining those findings with our present *in vivo* data, it seems reasonable to conclude that omental tissue-derived molecules elicited diverse genetic aberrations within gastric cancer cells and thus affected their biological behavior in mice without further interaction with omental tissue. In addition to the induction of tumor growth, our *in vivo* data showed a significant increase in tumor vasculature. We and others have shown that human omental tissue cells secrete a number of distinct pro-angiogenic factors (i.e., VEGF, IGF-1, etc.), thus enhancing angiogenesis both *in vitro* and *in vivo* (23).

To strengthen our results and to expand our findings, we characterized the omental tissue secretome. Our LC-MS/MS analysis provided a long list of distinct omental tissue proteins. We further analyzed these proteins according to their biological functions, focusing on potential processes related to gastric cancer tumorigenesis and metastasis. The attachment of cancer cells to the omentum which is mediated largely by adhesion molecules is a critical step in the formation of gastric cancer omental spread. Our analysis identified a group of seven proteins specifically associated with homing and binding/adhesion of tumor cells. Of them, the expression of the adhesion molecule FN1 was significantly increased. Interestingly, FN1 was reported to be elevated in omental-derived mesothelial cells treated with cancer exosomes during the development of peritoneal metastasis of gastric cancer (37). These data indicate that FN1 may play a critical role in the development of gastric cancer carcinomatosis and therefore should be further investigated within this context. In accordance with our phenotypic observations, we also showed that the omental secretome included numerous molecules which have an established role in cancer cell proliferation, cellular motility, metastasis, angiogenesis, and chemoresistance.

Finally, we sought to evaluate the potential role of exosomes in the omental tissue-gastric cancer crosstalk. Our preliminary data support the hypothesis that omental tissue-derived exosomes may promote gastric cancer progression and metastasis. In the present study, we show that exosomes can be purified from the CM of human omental tissue, that they are taken up by gastric cancer cells, and promote their proliferation and motility. We demonstrate that the internalization of omental tissue exosomes by gastric cancer cells is an active process which involves several endocytosis pathways mediated by heparan sulfate proteoglycans (HSPGs), dynamin tyrosine kinase and cholesterol. Since omental tissue exosomes originate from different cells, it is possible that their origin also dictates their route of internalization.

To characterize the content of these exosomes we performed a cytokine array which identified a number of candidate proteins plausibly related to omental- induced gastric cancer metastasis; of them, IL-6 and IL-8 were the most abundant. These two cytokines were also dominant in our omental secretome. Previous data have shown that serum IL-6 levels are significantly higher in GC patients with peritoneal metastasis (46). Additionally, both IL-8 and IL-6 which are produced by adipocytes and mesothelial cells were also reported to promote the peritoneal dissemination and metastasis of gastric cancer cells (49, 50). ICAM-1 has been implicated in adhesion of colorectal, pancreatic, and gastric cancer cells to the peritoneal mesothelium and the progression of carcinomatosis (47, 48). Our data analysis identified ample levels of ICAM-1 in omental tissue exosomes.

To date, data on adipose tissue-derived exosomes are generally related to obesity and metabolism (17–20). However, several reports have demonstrated their role in cancer (62–65). While the present study describes the potential role of omental- derived exosomes in the development and progression of gastric cancer peritoneal metastasis, others have focused on gastric cancer- derived exosomes. Deng et al. reported that gastric cancer-derived exosomes play a crucial role in remodeling the pre-metastatic microenvironment for peritoneal metastasis of gastric carcinoma by destroying the mesothelial barrier (66). Arita et al. have shown that gastric cancer exosomes enhance expression of adhesion molecules in mesothelial cells, thus promoting peritoneal metastasis (37). Tokuhisa et al. described the isolation of exosomes from malignant ascites and the intraoperative peritoneal lavage fluid samples obtained from patients with gastric cancer. Unrelated to their cellular origin, the authors concluded that specific molecules within these exosomes may enable early diagnosis of peritoneal dissemination of gastric cancer (67).

Taken together these data, we suggest that gastric cancer cells as well as omental tissue- derived exosomes induce numerous reciprocal molecular alternations within the metastatic niche enhancing homing, surviving, and thriving within the omental microenvironment.

In conclusion, our study demonstrated that the omentum promotes cellular aggressiveness in the setting of gastric cancer. This effect is probably paracrine and mediated by a large number of secreted proteins which induce different critical elements of cancer cell progression *in vitro* and *in vivo*. In addition, we showed that omental tissue-derived exosomes may also take part in the process of gastric cancer omental and potentially peritoneal spread. Further investigation is needed to identify the exact molecular pathways involved in that intriguing crosstalk between the omentum and gastric cancer cells. We believe that such research will improve our understanding of the peritoneal spread of gastric cancer and enable us to develop better therapies for this disease process.

## Data Availability Statement

All relevant data is contained within the manuscript.

## Ethics Statement

The studies involving human participants were reviewed and approved by Human Ethics Review Committee of the Israeli Ministry of Health and the Tel-Aviv Sourasky Medical Center. The patients/participants provided their written informed consent to participate in this study. The animal study was reviewed and approved by Institutional Animal Care and Usage Committee of the Sourasky medical center and the Israeli Ministry of Health.

## Author Contributions

SL, NL, and GL conceived and designed the project. OK, SL, and NS performed the experiments. OK, SL, OS, and GL analyzed and interpreted the data. OK, SL, GL, and JK wrote the manuscript. All authors contributed to the writing and reviewing of the manuscript, and approved the final manuscript for submission.

### Conflict of Interest

The authors declare that the research was conducted in the absence of any commercial or financial relationships that could be construed as a potential conflict of interest.

## Abbreviations

CM: conditioned medium
RM: regular medium
LC-MS/MS: liquid chromatography and tandem mass spectrometry
IHC: Immunohistochemistry.
